# Tangeretin Alleviates Cisplatin-Induced Acute Hepatic Injury in Rats: Targeting MAPKs and Apoptosis

**DOI:** 10.1371/journal.pone.0151649

**Published:** 2016-03-31

**Authors:** Hany A. Omar, Wafaa R. Mohamed, Hany H. Arab, El-Shaimaa A. Arafa

**Affiliations:** 1 Department of Pharmacology and Toxicology, Faculty of Pharmacy, Beni-Suef University, Beni-Suef 62514, Egypt; 2 Sharjah Institute for Medical Research, College of Pharmacy, University of Sharjah, Sharjah 27272, United Arab of Emirates; 3 Department of Biochemistry, Faculty of Pharmacy, Cairo University, Cairo 11562, Egypt; 4 Department of Pharmacology and Toxicology, Faculty of Pharmacy, Taif University, Taif 21974, Saudi Arabia; University of Calcutta, INDIA

## Abstract

Despite its broad applications, cisplatin affords considerable nephro- and hepatotoxicity through triggering inflammatory and oxidative stress cascades. The aim of the current investigation was to study the possible protective effects of tangeretin on cisplatin-induced hepatotoxicity. The impact of tangeretin on cisplatin-evoked hepatic dysfunction and histopathologic changes along with oxidative stress, inflammatory and apoptotic biomarkers were investigated compared to silymarin. Tangeretin pre-treatment significantly improved liver function tests (ALT and AST), inhibited cisplatin-induced lipid profile aberrations (total cholesterol and triglycerides) and diminished histopathologic structural damage in liver tissues. Tangeretin also attenuated cisplatin-induced hepatic inflammatory events as indicated by suppression of tumor necrosis factor-α (TNF-α) and enhancement of interleukin-10 (IL-10). Meanwhile, it lowered malondialdehyde (MDA), nitric oxide (NO) and nuclear factor erythroid 2-related factor 2 (NRF-2) levels with restoration of glutathione (GSH), and glutathione peroxidase (GPx). Regarding mitogen-activated protein kinase (MAPK) pathway, tangeretin attenuated cisplatin-induced increase in phospho-p38, phospho-c-Jun N-terminal kinase (p-JNK) and phospho-extracellular signal-regulated kinase (p-ERK1/2) in liver tissues. In addition, tangeretin downregulated Bax expression with augmentation of Bcl-2 promoting liver cell survival. Our results highlight the protective effects of tangeretin against cisplatin-induced acute hepatic injury via the concerted modulation of inflammation, oxidative stress, MAPKs and apoptotic pathways.

## Introduction

Cisplatin is one of the most widely used anticancer agents in the management of different malignancies. While 70–80% of patients respond to platinum treatment, such an initial effect is not robust, and results from a 5-year patient survival study revealed that the response is only 15–20% due to the development of resistance [1]. The relapse of the disease and the emergence of resistance in initially responsive tumors occur within 18–24 months [2, 3]. The dose scale necessary to overcome even a small increase in cellular resistance can lead to severe cytotoxicity in normal cells, such as nephrotoxicity, hepatotoxicity and spermiotoxicity which radically limits the clinical usefulness of cisplatin-based therapy [4–6]. Cisplatin-induces nephrotoxicity through multiple mechanisms, including hypoxia, the generation of free radicals, inflammation, and apoptosis with an increase in the pro-apoptotic protein Bax and a decrease in the anti-apoptotic protein Bcl-2 [7]. While, the mechanisms of cisplatin-induced hepatotoxicity are not fully understood [8], the link between oxidative stress and cisplatin toxicity was suggested in many experimental models [9, 10]. Several reports have implicated free radicals and reactive oxygen species (ROS) in cisplatin toxicity associated with an increase in lipid peroxidation (LPO), decreased levels of protein bound sulfhydryl groups and glutathione [11].

There is an increasing interest in the use of phytochemicals for evaluating their synergistic efficacy in combination with chemotherapeutic agents [12]. This is supported by the vast epidemiological data indicating the protective effect of vegetables and fruit intake rich in naturally occurring compounds against various diseases including cancer [13]. Flavonoids are dietary compounds that are widespread in fruits and vegetables. They have demonstrated a good potential as anticancer agents via their antiproliferative activity against human tumor cell lines [14], therefore, they were employed in cancer combination therapies for greater efficacy and safety [15].

Tangeretin, a citrus flavonoid concentrated in the peel of citrus fruits, has exhibited significant anti-inflammatory and antioxidant activities [16, 17]. Tangeretin oral bioavailability and safety have been reported previously [18, 19]. Notably, it has inhibited cancer cell proliferation in human cancer cell lines derived from squamous cell carcinoma, gliosarcoma, leukemia, melanoma, colorectal cancer, gastric carcinoma, lung carcinoma, breast carcinoma and oral cancer cells [20–22]. In a previous study from our laboratory, we have reported that pretreatment of cisplatin-resistant human ovarian cancer cells with tangeretin synergistically enhanced the growth inhibitory effects induced by low dose of cisplatin [23]. The combination markedly triggered apoptosis and arrested the cell cycle at G_2_-M. Additionally, the phosphoinositide3-kinase (PI3K)/protein kinase B (Akt) survival pathway was effectively downregulated. The fact that tangeretin enhances the cytotoxic actions of cisplatin encouraged us to investigate whether tangeretin, as a bioactive flavonoid, can protect against cisplatin-evoked hepatotoxicity, a serious complication of cisplatin that may limit its therapeutic utility. To this end, the hepatoprotective effects of tangeretin were assessed by measuring its ability to antagonize cisplatin-induced inflammation, oxidative stress and apoptotic cell death in rat liver. The effect of tangeretin was compared to silymarin, a naturally occurring flavonoid used as a reference hepatoprotective agent. Several studies have reported its hepatoprotective effects in acute and chronic hepatic diseases [24–26]. Silymarin has been reported to protect against CCl4- [27–29], acetaminophen- [30, 31], D-galactosamine- [32] and cisplatin- [33] induced liver injuries.

## Material and Methods

### Ethics Statement

The experimental protocol of the current study was approved by the Ethics Committee of the Faculty of Pharmacy, Beni-Suef University (Reference Number: 2014/A-32). The Guidelines for the Care and Use of Laboratory Animals declared by the US National Institute of Health were followed in all the experimental procedures.

### Animals

Adult male Wistar rats weighing 160–200 g were obtained from the National Institute for Research, Cairo, Egypt. Rats were kept under controlled conditions of temperature (22±1°c), humidity (60±10%) and normal photoperiod (12–12 h light-dark cycles) with free access to a standard commercial pellet diet and water.

### Materials

Cisplatin was purchased from Sigma-Aldrich, MD, USA, whereas tangeretin was purchased from Shaanxi Huike Botanical Development Co. (Xi'an, China). All other chemical reagents used in the study were of analytical grade (AR). Antibodies against Bax, Bcl-2 and β-actin were purchased from Santa Cruz Biotechnology (Santa Cruz, CA) while Erk1/2, p-Erk1/2, JNK, p-JNK, p38 and p-p38 antibodies were purchased from cell Signaling Technology (Beverly, MA).

### Experimental design and treatment protocol

The animals were divided into 7 groups, each with eight rats, according to their experimental treatment as follows: (**1**) Normal (control) group: rats received only oral vehicle (2% Tween 80) p.o for 7 consecutive days and a single i.p injection of isotonic saline on the 2^nd^ day of the experiment; (**2**) Silymarin group: rats received silymarin (100 mg/kg/day p.o) for 7 days and a single i.p injection of isotonic saline on the 2^nd^ day of the experiment; (**3**) Tangeretin group: received tangeretin (100 mg/kg/day p.o) for 7 days and a single i.p injection of isotonic saline on the 2^nd^ day of the experiment; (**4**) Cisplatin group: received oral vehicle for 7 days and a single dose of cisplatin (7.5 mg/kg i.p) on the 2^nd^ day of the experiment; (**5**) Cisplatin-Silymarin group: rats received silymarin (100 mg/kg/day p.o) for 7 days and a single dose of cisplatin (7.5 mg/kg i.p) on the 2^nd^ day of the experiment, 1 hour after the dose of silymarin; (**6**) Cisplatin-Tangeretin 50 group: rats received tangeretin (50 mg/kg/day p.o) for 7 days and a single dose of cisplatin (7.5 mg/kg i.p) on the 2^nd^ day of the experiment day, 1 hour after the dose of tangeretin; (**7**) Cisplatin-Tangeretin 100 group: rats received tangeretin (100 mg/kg/day p.o) for 7 days and a single dose of cisplatin (7.5 mg/kg i.p) on the 2^nd^ day of the experiment day, 1 hour after the dose of tangeretin.

The selected doses of cisplatin and tangeretin were chosen after performing preliminary experiments based on previous dose-response studies that have been reported to cause hepatotoxicity [33, 34] and marked antitumor effects in rats [17, 35].

### Tissue collection and preparation

Animals were sacrificed under ether anesthesia on the last day of the experiment. For the different biochemical measurements, blood samples were collected and allowed to stand for 30 min at 37°C, and then centrifuged at 1000 × g for 15 min at 4°C to separate serum and were stored at −70°C. The liver tissues were quickly harvested and one part of the liver tissue was instantly fixed in 10% phosphate buffered formaldehyde for histological and immunohistochemical studies. For the biochemical determinations, another part of liver was homogenized in lysis buffer containing protease and phosphatase inhibitor cocktails (Sigma-Aldrich, St. Louis, MD, USA).

### Serum Biochemical Tests

The collected serum was used for the colorimetric estimation of alanine aminotransferase (ALT) and aspartate aminotransferase (AST) as mentioned before [36] by measuring the amount of pyruvate or oxaloacetate produced by forming 2,4-dinitrophenylhydrazone. The produced color was measured spectrophotometerically at 546 nm. Concentrations of triglycerides (TGs) and serum total cholesterol (TC) were measured by enzymatic colorimetric methods using commercial kits (Spinreact, Gerona, Spain).

#### Oxidative Stress Biomarkers

Liver homogenates were used to determine nitric oxide (NO) essentially as mentioned before, using Nitric Oxide (NO_2_^-^/NO_3_^-^) assay Kit (Assay Designs, Ann Arbor, MI, USA) [37]. Greiss reagent was used for the quantitative colorimetric determination of NO levels as total nitrate/nitrite. To reduce nitrate to nitrite, vanadium trichloride and then Griess reagent was added and incubated at 37°C. The absorbance was measured after allowing the mixture to cool at 540 nm. Results were expressed as μmol/g tissue.

Glutathione (GSH) levels in liver tissues were, measured as mentioned before [38]. Briefly, after the precipitation of liver proteins by 10% trichloroacetic acid, 10 mM DTNB (5,5’- dithiobis 2-nitrobenzoic acid) solution was added to develop the color that was measured at 412 nm. Results were expressed as μmol/g tissue.

### Enzymatic and Transcriptional Antioxidant Status

The homogenates of liver tissues were utilized in the determination of glutathione peroxidase (GPx) activity using the corresponding assay kit (Sigma-Aldrich, St. Louis, MD, USA) according to the manufacturer’s guidelines. The decrease in the absorbance of NADPH was determined at 340 nm. The amount of enzyme which oxidizes one μmol of NADPH per min at 25°C is defined as one unit of enzyme.

Lipid peroxides in liver tissues were expressed as malondialdehyde (MDA) and determined as mentioned before [39]. The reaction proceeded in trichloroacetic acid, the precipitate was removed and the absorbance was measured at 535 nm. Results were presented as nmol/g tissue. The nuclear factor erythroid 2-related factor (NRF-2) is a transcription factor that controls the redox homeostatic gene network. Hepatic tissue NRF-2 was estimated using Total NRF-2 Cell-Based Colorimetric ELISA Kit (Immuno Way Biotechnology, Newark, DE, USA) as directed by the manufacturer.

### Histopathologic Examination

Samples of tissues were fixed in 10% neutral formalin for 24 h, and paraffin blocks were then processed for light microscopy examination. Slices of 4–5 μm were obtained from the prepared blocks and stained with hematoxylin-eosin (H&E). The preparations obtained were visualized using a Nikon microscopy at a magnification of 400×.

### Immunohistochemistry of Bax and Bcl-2 expression

Antigen retrieval and immunohistochemistry was performed essentially as mentioned before [40]. In brief, paraffin-embedded tissue samples were rehydrated then blocked by 5% bovine serum albumin (BSA) in Tris buffered saline. The samples were then incubated overnight at 4°C with primary antibodies against Bax or Bcl-2 (Santa Cruz Biotechnology Inc, CA, USA). The slides were then washed and incubated with secondary antibodies. The sections were then washed and visualized using 3,3’-diaminobenzidine tetrahydrochloride (DAB Horseradish Peroxidase Substrate Kit, Vector Laboratories Inc, Burlingame, CA, USA). Counter staining with hematoxylin was used and the slides were observed under a light microscope (Leica Microsystems, Germany) by an experienced observer blinded to the identity of the sample.

### Western blotting

Liver tissue homogenates were prepared for Western blotting as mentioned before [41]. Briefly, protein concentration in the tissue lysate was determined using DC protein assay kit (Bio Rad). Protein samples were then separated by SDS-PAGE (30 μg per lane) and transferred to a nitrocellulose membrane. The membrane were blocked using 5% (w/v) non-fat dry milk in Tris buffered saline-tween 20 (0.025 M Tris; 0.15 M NaCl; 0.05% Tween 20; pH 7.4), incubated overnight at 4°C with primary antibodies, rinsed, and then incubated with horseradish peroxidase-conjugated secondary antibodies (Santa Cruz Biotechnology, Santa Cruz, CA) for 1 h at room temperature before the detection using Super Signal West Pico chemiluminescent substrate (Pierce, Rockford, IL). β-actin was used as a loading control. Band density in intermediately exposed films was quantitated using ImageJ image processing (ImageJ, National Institutes of Health, USA).

### Caspase 3/7 assay

Caspase-3/7 activities in liver tissue homogenate were measured using a Caspase-Glo assay kit (Promega, Madison, WI, USA) following the manufacturer’s instructions.

### Statistical analysis

The data were expressed as mean ± SEM. Statistical analysis was done using one-way analysis of variance (ANOVA), followed by Tukey-Kramer post hoc multiple comparisons among treatment means. The analysis was done using SPSS program, version 17. Differences were considered significant at p < 0.05.

## Results

### Tangeretin pre-treatment improves liver function and inhibits cisplatin-induced aberrations in lipids profile

To assess the severity of cisplatin-induced liver injury, liver function tests were performed. Results revealed that the used dose of cisplatin triggered severe liver injury as indicated by elevated serum ALT and AST enzyme activities. Meanwhile, increased serum total cholesterol and triglyceride in cisplatin-treated group was observed showing an extensive damage to the liver tissues (Table 1).

**Table 1:** Tangeretin pre-treatment improved liver function and inhibited cisplatin-induced aberrations in lipids profile.

	ALT (U/l)	AST (U/l)	Triglyceride (mg/dl)	Total Cholesterol (mg/dl)
**Normal**	24.5 ± 1.7	112.3 ± 13.6	53.3 ± 3.5	72.3 ± 4.9
**Silymarin (100 mg/kg)**	25.5 ± 3.1	112.5 ± 12.5	57.5 ± 5.2	69.5 ± 8.2
**Tangeretin (100 mg/kg)**	27.5 ± 2.4	114.3 ± 17.2	62.2 ± 8.4	71.6 ± 4.2
**Cisplatin**	77.3 ± 7.0*	290.0 ± 38.3*	175.2 ± 19.7*	138.0 ± 12.8*
**Cisplatin + Silymarin (100 mg/kg)**	38.8 ± 4.6^#^	136.2 ± 12.7^#^	111.0 ± 12.5^#^	89.6 ± 10.5^#^
**Cisplatin + Tangeretin (50 mg/kg)**	68.0 ± 5.1	252.8 ± 36.1	159.7 ± 20.2	122.4 ± 5.4
**Cisplatin + Tangeretin (100 mg/kg)**	35.8 ± 4.2^#^	132.2 ± 12.3^#^	84.8 ± 13.1^#^	82.5 ± 6.3^#^

Values are mean± SD of the mean (n = 4–5 independent values). Statistical analysis was carried out by using one way analysis of variance (ANOVA) followed by Tukey-Kramer multiple comparisons test

* Significantly different from normal control at p < 0.05.

^**#**^ Significantly different from cisplatin-treated (7.5 mg/kg) animals at p < 0.05.

On the other hand, pretreatment with tangeretin for 1 week significantly alleviated liver damage as evidenced by a dose-dependent restoration of the normal liver functions and lipid profile. At the same time, the reference hepatoprotective, silymarin afforded remarkable hepatic protection against cisplatin-induced liver injury. These data suggest the hepatoprotective activity of tangeretin against the development of cisplatin-induced liver tissue damage which were similar to that obtained by silymarin (Table 1).

### Tangeretin attenuates liver histopathological aberrations

**Fig 1:**
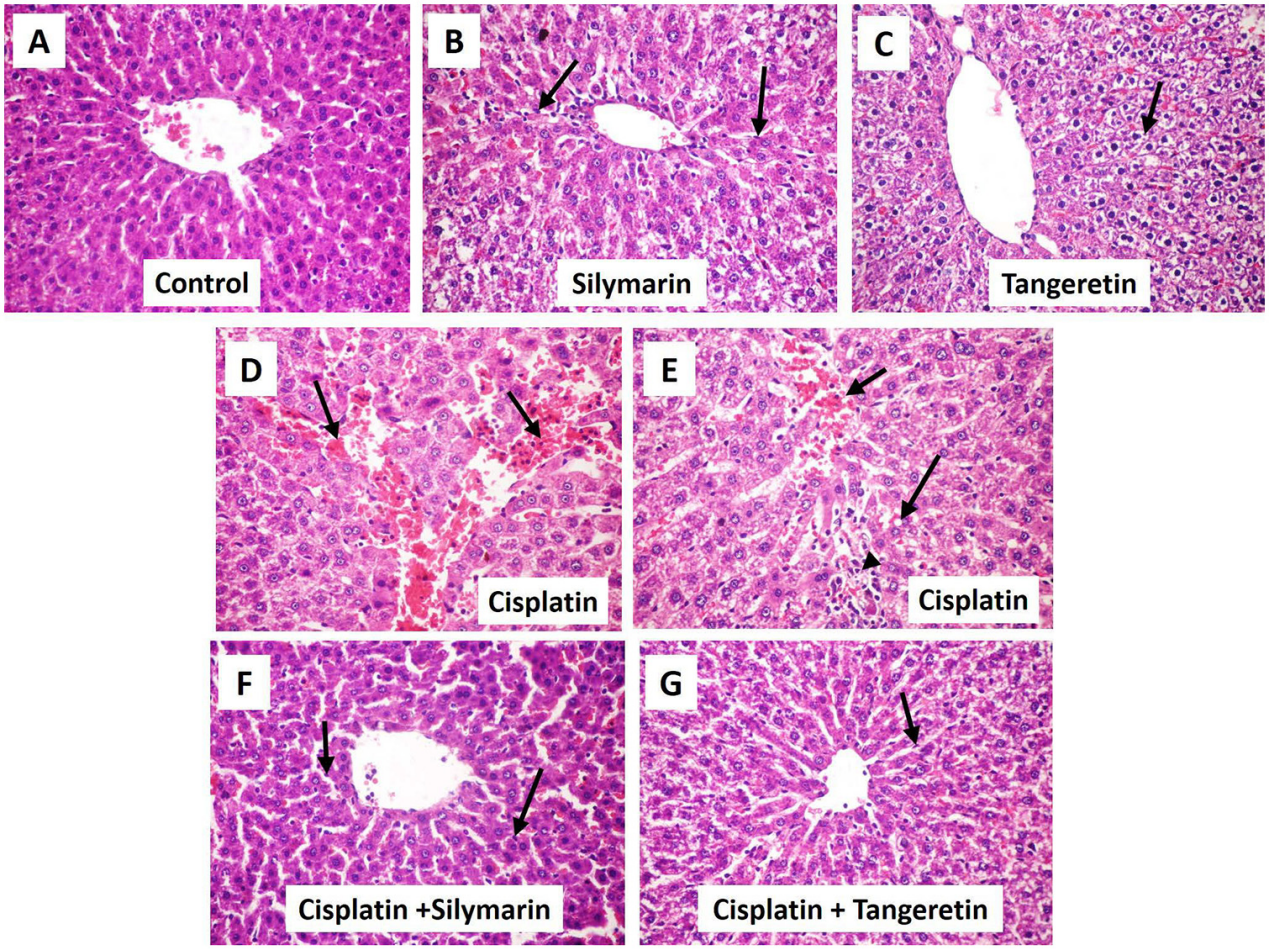
Tangeretin alleviates cisplatin-induced hepatic histopathologic injury in rats. Representative photomicrographs of sections from of liver tissues stained by hematoxylin and eosin (× 400 magnification). (**A**) Control rats receiving saline vehicle showed normal histological structure of the hepatic lobule. (**B**) Rats which received silymarin (100 mg/kg p.o.) showed normal hepatic histology with few Kupffer cell activation (arrow). (C) Rats which received tangeretin (100 mg/kg p.o.) showed slight hydropic degeneration of hepatocytes (arrow). (**D**) Cisplatin-treated group showed dilatation and congestion of central vein and hepatic sinusoids (arrow). (**E**) Liver of rat from cisplatin-treated group group demonstrated congestion of central vein (short arrow), cytoplasmic vacuolization of hepatocytes (long arrow) and focal hepatic necrosis associated with inflammatory cell infiltration (arrow head) (**F**, **G**) Silymarin and tangeretin pre-treatment revealed attenuated morphological modifications with resolving Kupffer cell activation (arrow).

We next explored the ability of tangeretin to protect against the histopathologic changes accompanying cisplatin-induced liver injury. Liver sections of control, silymarin and tangeretin groups showed the normal architecture of the liver tissues with minor Kupffer cells activation (Fig 1). On the other hand, the administration of cisplatin caused a severe liver injury (Table 2), as reflected by the dilatation and congestion of central vein and hepatic sinusoids with Kupffer cell activation and focal hepatic necrosis. This was accompanied with diffuse inflammatory cell infiltration. Pretreatment with tangeretin decreased the high pathologic changes, suggesting the attenuation of liver damage with the preservation of the liver wall architecture. These effects were closely analogous to those afforded by silymarin (Fig 1).

**Table 2:** Liver microscopic damage.

Histopathological alterations	Control	Silymarin	Tangeretin	Cisplatin	Cisplatin+ Silymarin	Cisplatin + Tangeretin
Kupffer cells activation	**-**	**+**	**-**	**++**	**+**	**+**
Congestion of central vein and hepatic sinusoids	**-**	**-**	**-**	**+++**	**+**	**-**
Cytoplasmic vacuolation of hepatocytes	**-**	**-**	**-**	**++**	**-**	**+**
Focal hepatic necrosis associated with inflammatory cell infiltration	**-**	**-**	**-**	**++**	**-**	**-**
Hydropic degeneration of hepatocytes	**-**	**-**	**+**	**-**	**-**	**-**

+++ Extensive

++ Moderate

+ Mild

–Nil

### Tangeretin attenuates cisplatin-induced inflammatory response and apoptosis in hepatic tissues

**Fig 2:**
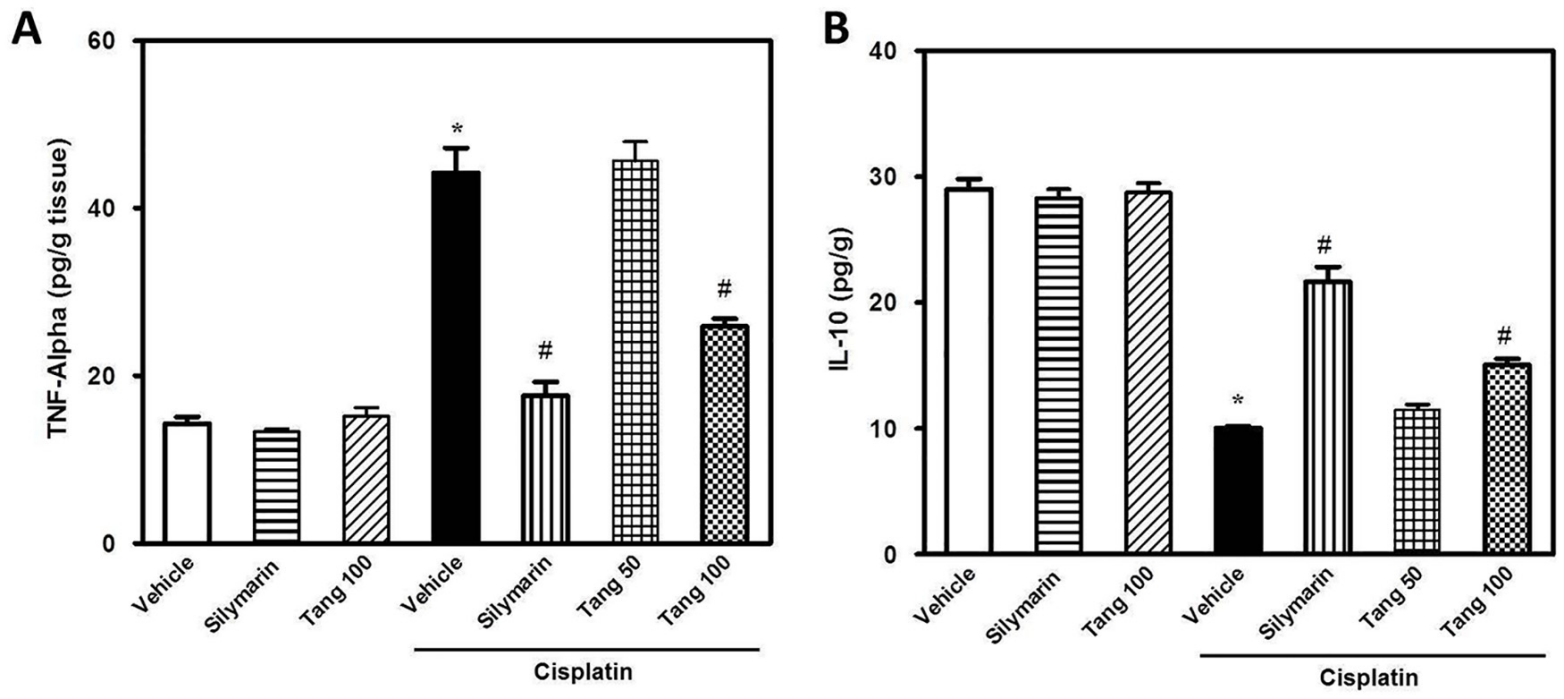
Tangeretin mitigates cisplatin-induced inflammatory response in liver tissues. The effect of tangeretin pretreatment on cisplatin-induced inflammatory response in liver tissues as indicated by the modulation of TNF-α (A) and IL-10 (B). Columns, mean; bars, ± SEM (n = 8 independent values). Statistical analysis was carried out by using one way analysis of variance (ANOVA) followed by Tukey-Kramer multiple comparisons test. *Significant difference from normal control (vehicle) group at p < 0.05, ^#^Significant difference from cisplatin group at p < 0.05. Tang 50; tangeretin (50 mg/kg), Tang 100; tangeretin (100 mg/kg).

**Fig 3:**
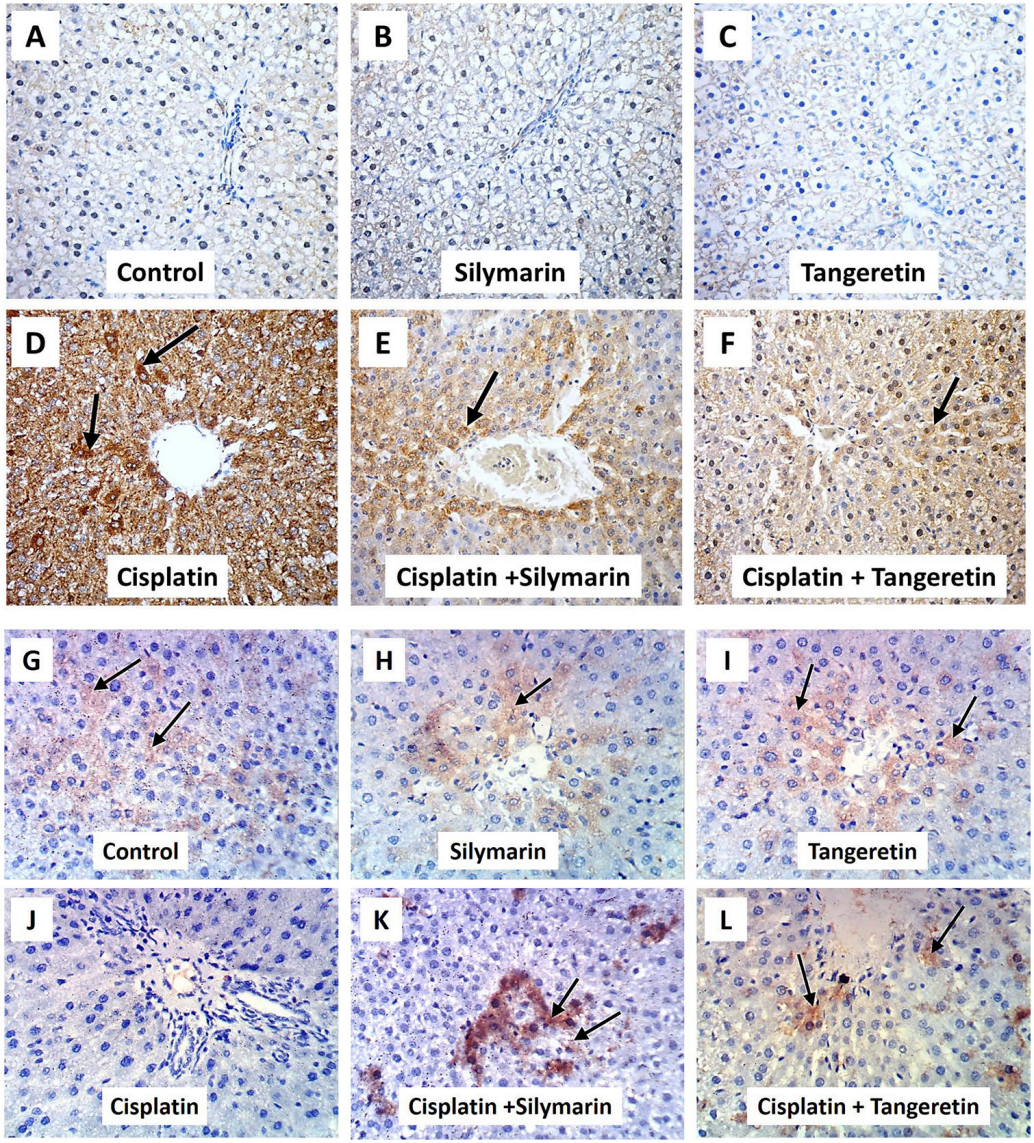
Tangeretin modulates cisplatin-induced protein expression of Bax and Bcl-2 in rat liver. Representative images for the immunohistochemical detection of Bax (**A -F**) and Bcl-2 (**G-L**) expression in liver tissues (arrows) (magnification: × 400).

**Fig 4:**
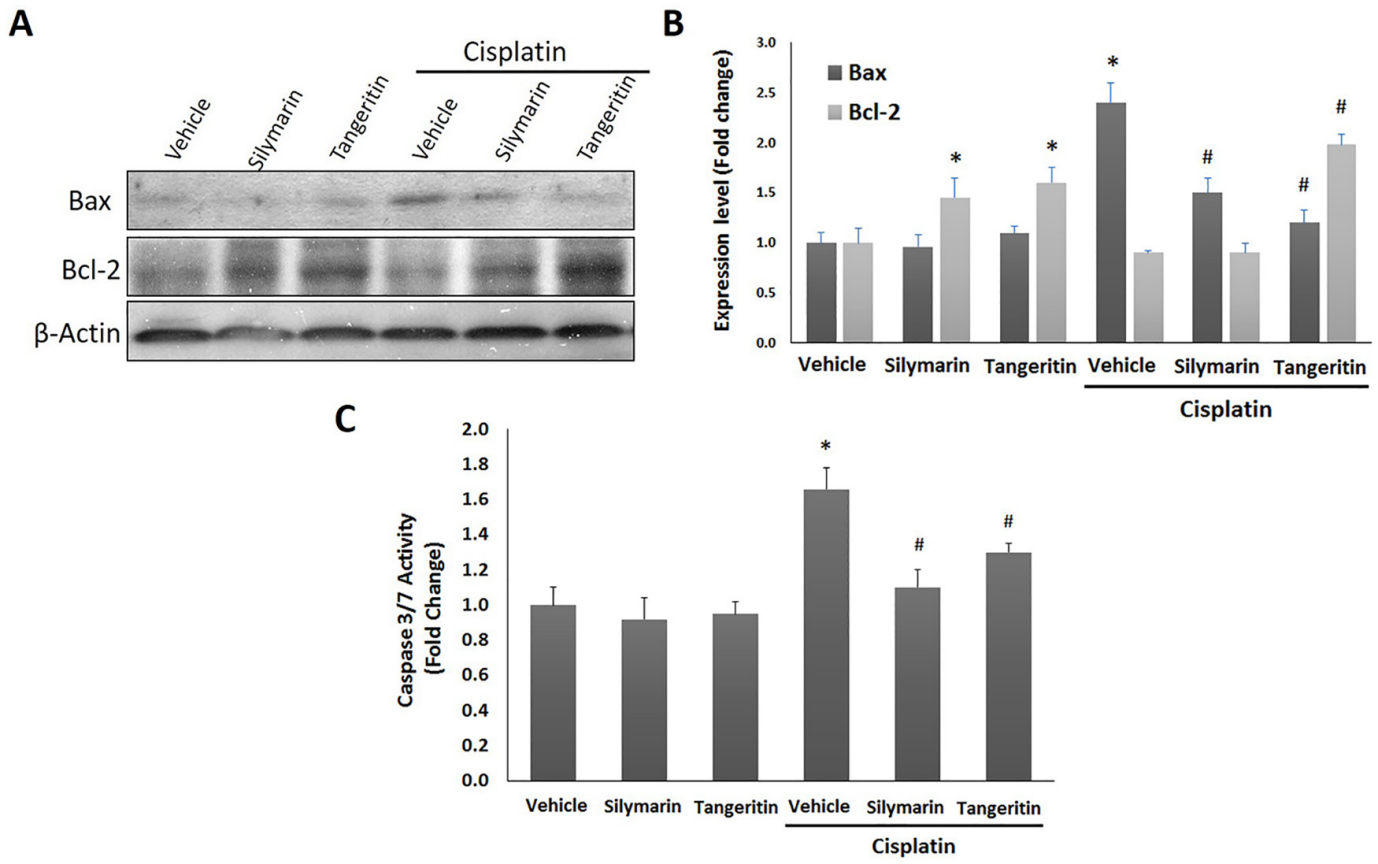
Tangeretin counteracts cisplatin-induced apoptotic changes of Bax and Bcl-2 in protein expression in rat liver. (**A**) Western blot analysis showed the expression levels of Bax and Bcl-2 in liver tissues after the indicated treatments. (**B**) Relative expression levels of Bax and Bcl-2. The amount of immunoblotted proteins was quantitated by densitometry and normalized to that of β-actin. Columns, mean; bars, ± SD (n = 3 independent experiments). (**C**) Relative caspase 3/7 activity in liver tissues after the indicated treatments. Columns, mean; bars, ± SD (n = 4 independent values). Statistical analysis was carried out by using one way analysis of variance (ANOVA) followed by Tukey-Kramer multiple comparisons test. Silymarin (100 mg/kg); Tangeretin (100 mg/kg); Cisplatin (7.5 mg/kg). *Significant difference from normal control (vehicle) group at p < 0.05, ^#^Significant difference from cisplatin group at p < 0.05.

Cisplatin administration caused an inflammatory response as demonstrated by a 3.1 fold increase of hepatic TNF-α and a decline in IL-10 (32%) levels (Fig 2). Pretreatment with tangeretin at 100 mg/kg dose decreased the levels of TNF-α by 46% and slightly increased the IL-10. These results were similar to silymarin. In addition, the immunohistochemical detection of Bax and Bcl-2 revealed an extensive expression of Bax (Fig 3A–3F) and decreased Bcl-2 (Fig 3G–3L) in the hepatic tissues of rats treated with cisplatin; events which were markedly mitigated by administration of tangeretin. These results were confirmed by Western blot analysis of Bax and Bcl-2 (Fig 4A and 4B) and caspase 3/7 activation (Fig 4C) which indicated the ability of tangeretin to attenuate cisplatin-induced apoptosis in hepatic tissues. Analogous to silymarin, tangeretin counteracted these changes in favor of cell survival. Together, these observations suggested the modulation of inflammatory cytokines and suppression of apoptosis as crucial events in tangeretin protection against cisplatin-induced hepatic insult.

### Tangeretin limits oxidative stress and boosts hepatic antioxidant defense

**Fig 5:**
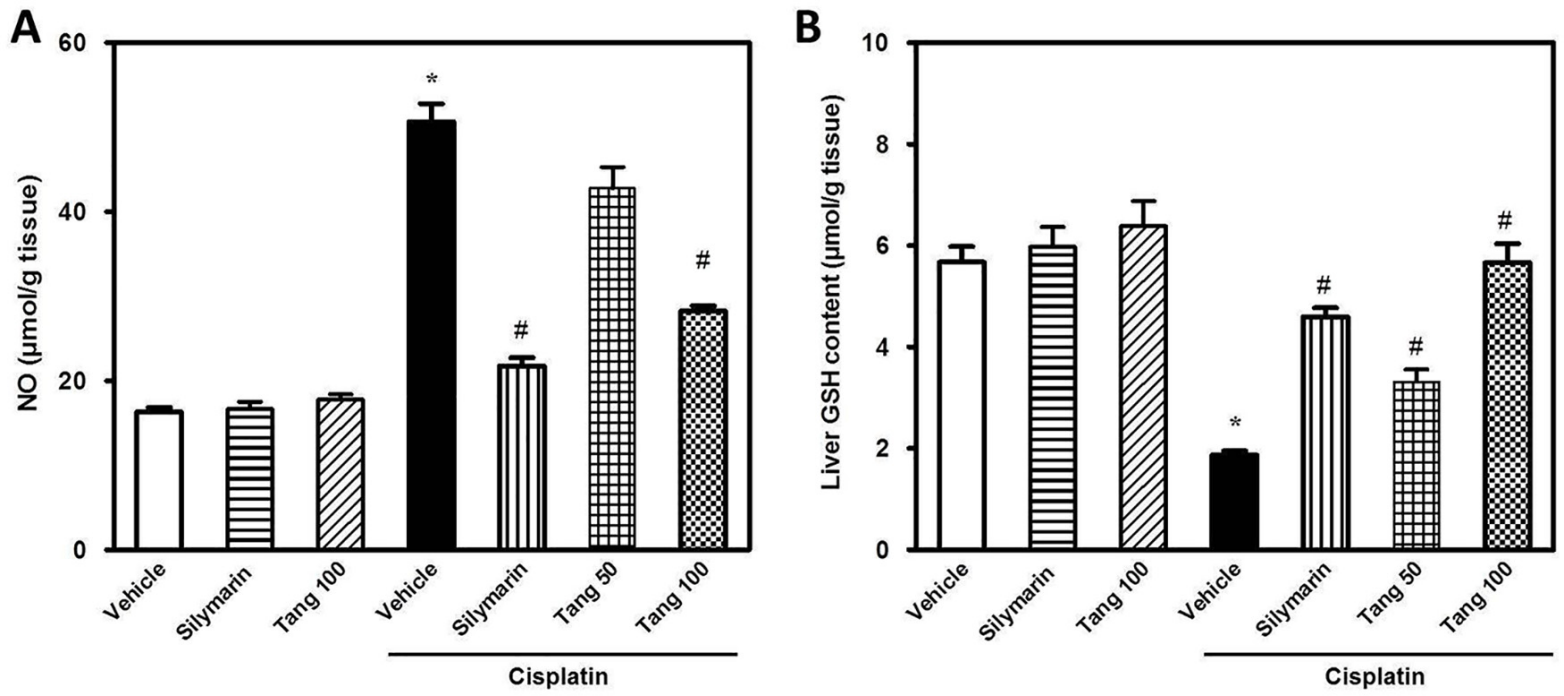
Tangeretin limits cisplatin-induced oxidative stress in liver tissues. The effect of tangeretin pretreatment on cisplatin-induced oxidative stress in liver tissues as indicated by the modulation of hepatic NO (A) and GSH content (B). Columns, mean; bars, ± SEM (n = 8 independent values). Statistical analysis was carried out by using one way analysis of variance (ANOVA) followed by Tukey-Kramer multiple comparisons test. *Significant difference from normal control (vehicle) group at p < 0.05, ^#^Significant difference from cisplatin group at p < 0.05. Tang 50; tangeretin (50 mg/kg), Tang 100; tangeretin (100 mg/kg).

**Fig 6:**
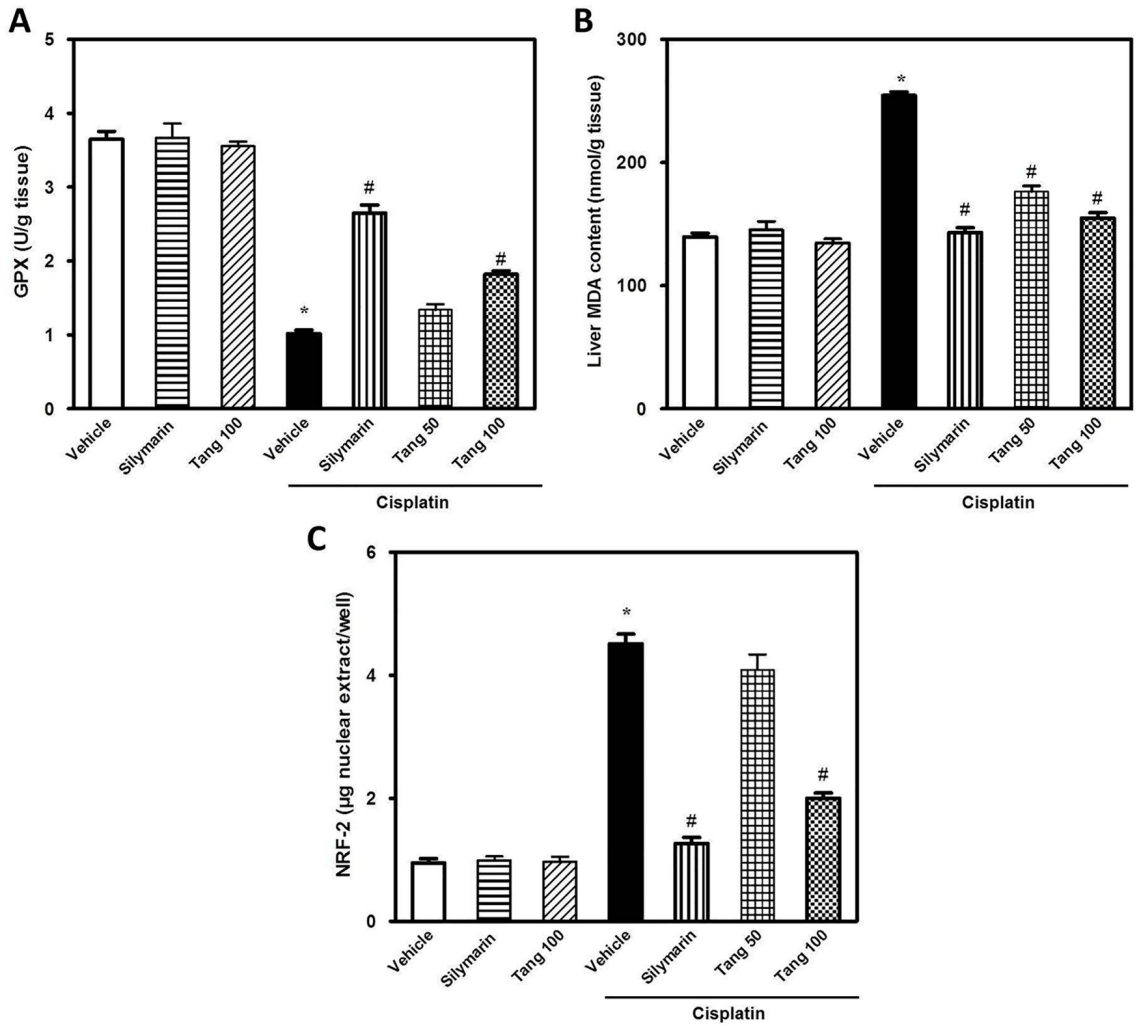
Tangeretin boosts hepatic antioxidant defense in cisplatin-induced oxidative stress in liver tissues. The effect of tangeretin pretreatment on cisplatin-induced oxidative stress in liver tissues as indicated by the modulation of GPx (A), MDA (B) and NRF-2 (C) content. Columns, mean; bars, ± SEM (n = 8 independent values). Statistical analysis was carried out by using one way analysis of variance (ANOVA) followed by Tukey-Kramer multiple comparisons test. *Significant difference from normal control (vehicle) group at p < 0.05, ^#^Significant difference from cisplatin group at p < 0.05. Tang 50; tangeretin (50 mg/kg), Tang 100; tangeretin (100 mg/kg).

Cisplatin administration elicited hepatic oxidative stress as demonstrated by elevation of NO levels (195%) and decline of GSH content (29%) (Fig 5). In addition, cisplatin caused a marked decrease of hepatic antioxidant defenses as indicated by the decrease in GPx activity (29%) together with elevation of MDA (210%) and NRF-2 (375%) as compared to the control group (Fig 6). Pretreatment with tangeretin significantly protected against the oxidative stress as indicated by lowering of MDA, NO and NRF-2 in addition to the restoration of GSH and GPx, events which were analogous to the actions of silymarin. Together, these data suggest that the ability of tangeretin to counteract cisplatin-induced oxidative stress and enhance the antioxidant defenses plays a role in the defense against cisplatin-induced hepatic injury.

### Tangeretin downregulates MAPK pathway in cisplatin-induced acute hepatic injury

**Fig 7:**
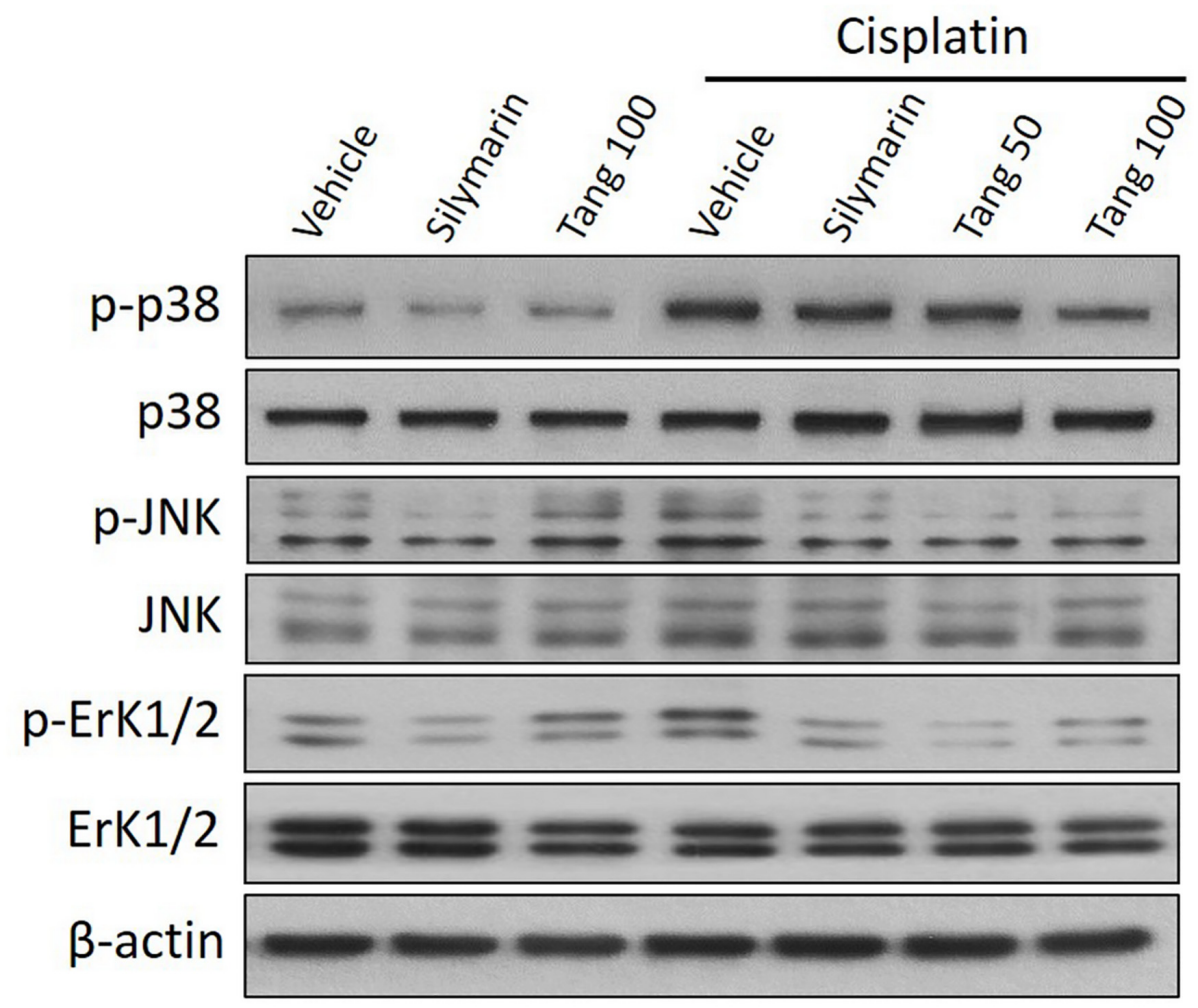
Tangeretin downregulates MAPK pathway in cisplatin-induced acute hepatic injury. Western blot analysis demonstrates that cisplatin increased the phosphorylation of p38 MAPK (upper panel), JNK (middle panel) and ErK1/2 (lower panel) without affecting the corresponding total protein levels, compared to the control group. Tangeretin and Silymarin pretreatments mitigated the phosphorylation of the three MAPKs. Tang 50; tangeretin (50 mg/kg), Tang 100; tangeretin (100 mg/kg).

The MAPK pathway has been reported to be activated in cisplatin-induced hepatic cytotoxicity [42]. Western blotting analysis revealed that administration of cisplatin activated MAPK signal transduction as evidenced by increased phosphorylation of p38 MAPK, JNK and ErK1/2 without affecting the corresponding total protein levels compared to the control group (Fig 7). Pretreatment with tangeretin attenuated the phosphorylation of the three hepatic MAPKs; effects which were comparable to those afforded by silymarin. Together, these data suggest that tangeretin downregulation of MAPK pathway plays a role in alleviating cisplatin-induced hepatic insult.

## Discussion

The present study sheds light on the possible protective actions of tangeretin, a natural flavone found in citrus peels, against cisplatin-induced hepatic injury in rats. In a previous study, we have addressed the ability of tangeretin to synergize the anticancer activity of cisplatin in ovarian cancer cell lines study through targeting cancer cell survival pathways such as phosphoinositide 3-kinase/Akt signaling [23]. The focus of the current study was whether tangeretin can alleviate cisplatin-induced hepatotoxicity, one of the serious complications of cisplatin that may limit its therapeutic utility and to move a step towards the translational application of tangeretin in cancer combinatorial protocols. Notwithstanding its valuable applications as an anticancer agent, cisplatin has significant nephrotoxic and hepatotoxic side effects. Cisplatin inflicts hepatic injury through the activation of inflammatory and oxidative stress pathways along with associated apoptosis and anomalies in liver structure and function [9, 10]. These complications besides the chemoresistance are the most important limiting factors for the clinical application of cisplatin in the cancer treatment [10, 43].

TNF-α and IL-10 play a crucial role in mediating the interplay between inflammatory, oxidative stress and apoptotic pathways [44–46]. Tangeretin afforded significant protection against cisplatin-induced hepatic injury in rats mainly through the suppression of TNF-α and upregulation of IL-10 and indirectly via combating ROS production. In addition, tangeretin protected against cisplatin effect through its anti-apoptotic effects which were similar to those afforded by the reference hepatoprotective, silymarin. These actions signify the potential use of tangeretin in attenuating cisplatin-induced liver lesions.

Cisplatin induces a massive inflammatory response in liver tissues which was observed by the elevation of the proinflammatory, TNF-α and the decline of the anti-inflammatory, IL-10 [47, 48]. TNF-α intensifies the hepatic tissue inflammation via the chemotaxis of immune cells and activation other cytokines [49]. On the other hand, the decline in IL-10 exacerbates hepatic lesion because of its ability to downregulate antigen presentation and the pro-inflammatory cytokines release [50, 51].

Tangeretin significantly inhibited TNF-α and restored IL-10 levels, which can be assumed as a chief mechanism for its hepatoprotective role in cisplatin-induced hepatic injury. The findings were parallel to the histopathologic results that showed the ability of tangeretin to attenuate the inflammatory cell infiltration and hepatic necrosis. The anti-inflammatory actions of tangeretin are mediated through the modulation of TNF-α and other inflammatory mediators [52].

Cisplatin administration triggered lipid peroxide formation and depleted the hepatic GSH and GPx. Oxidative stress was reported to be involved in cisplatin-induced acute hepatic injury [9, 10]. The depletion of GSH and GPx, which play major roles in the cellular defense against oxidative stress and cellular damage, renders hepatic tissues more susceptible to oxidative stress [53, 54]. Tangeretin administration alleviated oxidative stress and improved the antioxidant status in rats which was in agreement with previous studies [55, 56]. The observed antioxidant actions of tangeretin play a role in the protection against cisplatin injury.

Cisplatin induced apoptotic cell death in hepatic tissues by the modulation of Bax and Bcl-2 expression levels [57]. The current data revealed that tangeretin inhibited the pro-apoptotic Bax and increased the anti-apoptotic Bcl-2, indicating diminished hepatic apoptosis. The attenuation of hepatic tissue apoptosis can be related to the observed suppression of oxidative stress and TNF-α which enhance liver tissue apoptosis [58].

Cisplatin-induced hepatotoxicity has been reported to be associated with MAPK signal transduction pathway which plays a major role in mediating cellular inflammatory response and apoptosis [59, 60]. Our results revealed the ability of cisplatin to induce the phosphorylation of p38, JNK and ERK1/2 in liver tissues which are in accordance with previous reports [59]. These changes were abrogated by pretreatment with tangeretin which underlie the modulation of MAPK signaling pathway as a putative mechanism for the protection against cisplatin injury. This action could be secondary to its antioxidant and anti-inflammatory activities. The reactive oxygen species generated by cisplatin treatment can activate various downstream proteins that mediate apoptosis and necrosis, in particular, MAPK family proteins [61]. The MAPK family comprises of three major serine/Threonine kinase proteins such as ERK, JNK and p38 which are associated with cell growth and differentiation, and are extensively linked to inflammation, apoptosis and cell death [62]. Cisplatin treatment shifts the balance between pro- and anti-apoptotic signals towards proapoptotic cascade [63]. It affords upregulation of Bax, a proapoptotic effector and diminishes Bcl-2, an anti-apoptotic protein. It also induces translocation of Bax from cytosol to mitochondria releasing cytochrome c to cytosol [64]. Cytochrome c further activates caspase 8, 9 and ultimately 3, thereby triggering apoptotic cell death [65]. Previous reports have regarded caspase activation as a crucial cellular mechanisms for induction of apoptosis in renal tubular cells in cisplatin-induced acute renal injury [66]. Many *in vitro* and *in vivo* studies have demonstrated the central role of p38, JNK and ERK1/2 in cisplatin-induced oxidative stress and apoptosis [65]. It’s noteworthy that due to the mutual interplay between MAPKs and ROS with consequent deleterious effects on liver tissues, the current study cannot specifically confirm the activation of MAPK being a cause or a result for the hepatoprotection [67, 68].

In the same context, evidence has indicated that cisplatin treatment is associated with reactive oxygen species (ROS) generation which activates p38 MAPK resulting in apoptotic cell death in rat kidney [69]. The cross-talk between p38 MAPK and caspase signaling cascade was also reported in cisplatin-treated carcinoma and rat renal tissues [69]. It has been suggested that p38 and JNK favor apoptotic cell death [70, 71]. It is also reported that JNK is associated with TNF-α-induced apoptosis [72]. The interplay between p38 MAPK and TNF-α has been described in cisplatin-evoked renal injury [69] where p38 MAPK acts as an upstream signal which activates the transcription factor NF-κB with the consequent generation of TNF-α [73]. Abrogation of ROS production/ p38 MAPK activation by the antioxidants such as N-acetyl cysteine has considerably protected the renal tissues from cisplatin insult [69].

**Fig 8:**
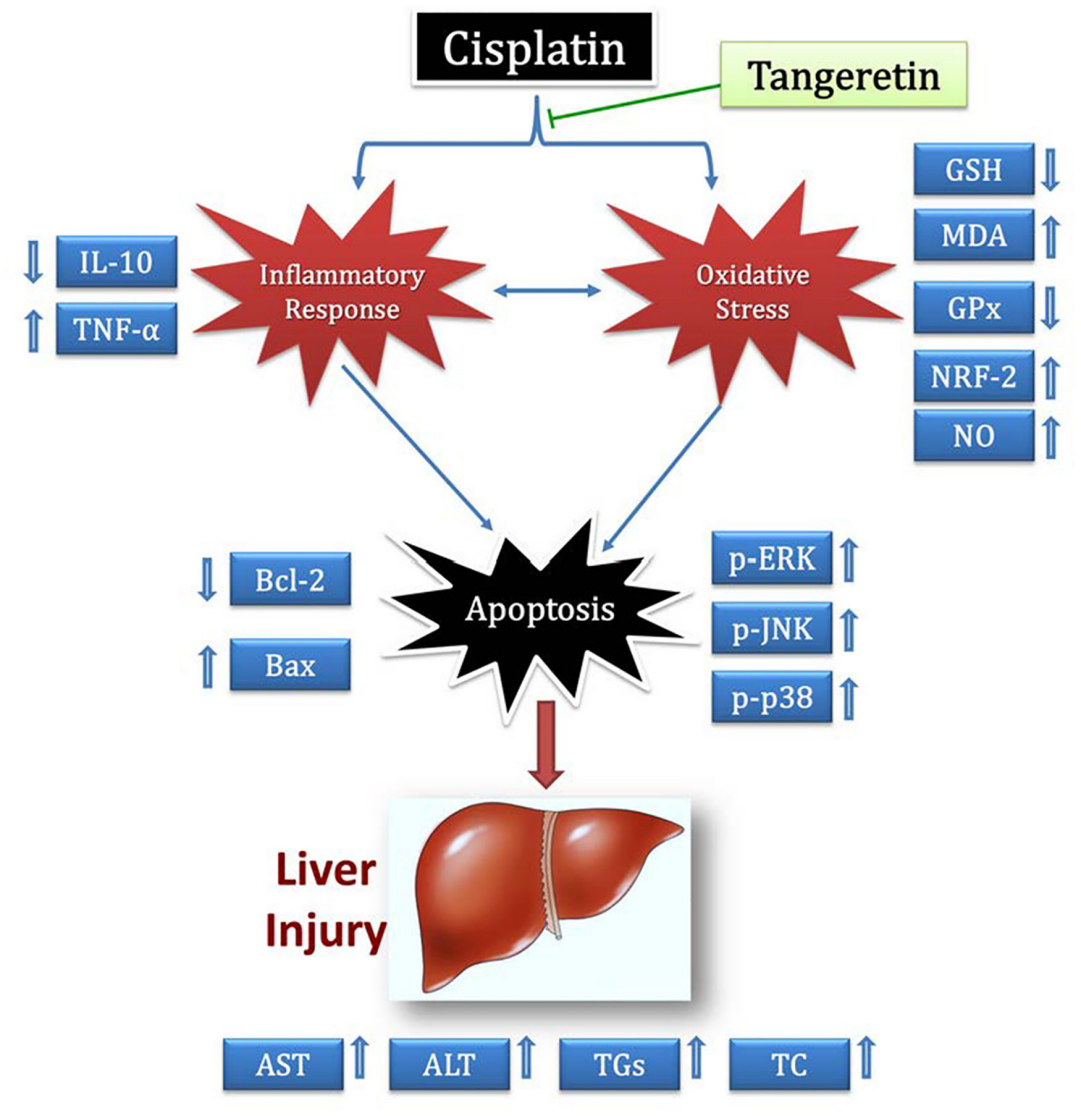
Diagram depicting the proposed protective mechanisms of tangeretin against cisplatin-induced hepatic injury.

In conclusion, the present work underscores the possible protective effects of tangeretin against cisplatin-induced acute hepatic injury in rats (Fig 8). These favorable actions confirm tangeretin benefits as an effective and safe approach for the management of one of cisplatin drawbacks. Further studies addressing the impact of tangeretin after repeated administration of cisplatin and at different time points are warranted, in order to delineate the exact underlying molecular mechanisms of tangeretin in cisplatin-evoked hepatotoxicity and the implication of other cell signaling networks.

## Abbreviations

ALT: alanine aminotransferase
AST: aspartate transaminase
GPx: glutathione peroxidase
GSH: glutathione
H&E: hematoxylin and eosin
IL-10: interleukin-10
MDA: malondialdehyde
NO: nitric oxide
NRF-2: nuclear factor erythroid 2-related factor 2
ROS: reactive oxygen species
TNF-α: tumor necrosis factor-α
